# Correlations between cardiac troponin I and nonsustained ventricular tachycardia in hypertrophic obstructive cardiomyopathy

**DOI:** 10.1002/clc.23425

**Published:** 2020-08-18

**Authors:** Limin Liu, Shangyu Liu, Lishui Shen, Bin Tu, Zhicheng Hu, Feng Hu, Lihui Zheng, Ligang Ding, Xiaohan Fan, Yan Yao

**Affiliations:** ^1^ Clinical EP Lab & Arrhythmia Center, Fuwai Hospital, National Center for Cardiovascular Diseases, State Key Laboratory of Cardiovascular Disease Chinese Academy of Medical Sciences and Peking Union Medical College Beijing China

**Keywords:** cardiac troponin I, hypertrophic obstructive cardiomyopathy, nonsustained ventricular tachycardia, sudden cardiac death

## Abstract

**Background:**

Nonsustained ventricular tachycardia (NSVT) is an independent risk factor for sudden cardiac death (SCD) in patients with hypertrophic obstructive cardiomyopathy (HOCM). However, data concerning the correlations of cardiac biomarkers and NSVT in HOCM are rather limited.

**Hypothesis:**

Our study aimed to investigate the associations between the occurrence of NSVT and circulating biomarkers representing myocardial injury (cardiac troponin I, cTnI), cardiac function (N‐terminal pro‐brain natriuretic peptide, NT‐pro BNP), and inflammation (high‐sensitivity C‐reactive protein) in a large Chinese HOCM cohort.

**Methods:**

A total of 755 consecutive HOCM patients were recruited. Systematic cardiac evaluations and circulating biomarkers were examined routinely in all subjects under the clinically stable status. According to the results of 24‐hour Holter monitoring, patients were divided into the NSVT group (n = 138) and the nonventricular tachycardia (non‐VT) group (n = 617).

**Results:**

Compared with the non‐VT group, circulating levels of both cTnI and NT‐pro BNP elevated significantly in patients with positive NSVT episodes (*P* < .001). Multivariable analyses demonstrated that cTnI was independently associated with the presence of NSVT (OR = 1.675, 95% CI: 1.406‐1.994, *P* < .001). Concentrations of cTnI increased progressively not only with the aggravation of ventricular arrhythmic events (*P* < .001), but also with the growing risk of SCD in HOCM patients (*P* < .001). Serum cTnI ≥ 0.0265 ng/mL indicated predictive value for the occurrence of NSVT in the HOCM cohort (area under the curve = 0.707, 95% CI: 0.660‐0.754, *P* < .001).

**Conclusions:**

Elevated cTnI was an independent determinant of NSVT, and it seemed to be valuable for assessing the clinical status of ventricular arrhythmias and the risk of SCD in patients with HOCM.

## 1 INTRODUCTION

Hypertrophic cardiomyopathy (HCM) is the most common inheritable cardiovascular disorder, with an estimated prevalence of 0.2% in the general population.^1^ It is mainly caused by mutations in genes encoding for sarcomeres, and confers a nearly 1% annual mortality rate^2^ linked to the complications of progressive heart failure, embolic stroke, and sudden death. Asymmetric septal hypertrophy constitutes the majority of HCM phenotypes, and approximately 70% of patients suffer from the associated obstruction of the left ventricular outflow tract (LVOT),^3^ referred to as hypertrophic obstructive cardiomyopathy (HOCM).

HCM is an important cause of sudden cardiac death (SCD), especially in young individuals.^4^, ^5^, ^6^ The European Society of Cardiology (ESC) 2014 guideline on HCM has suggested an easily applicable risk prediction model for estimating the 5‐year risk of SCD in HCM patients.^7^ Nonsustained ventricular tachycardia (NSVT) is demonstrated as an independent risk factor for SCD and has been included in the current HCM risk‐SCD scoring model. As a common arrhythmia in HCM, the detection rate of NSVT is approximately 20% to 35% using 24‐ to 48‐hour Holter monitoring.^8^, ^9^ The presence of NSVT episode, independent of its frequency, duration, or rate, carries a 2.8 relative risk of SCD compared to subjects without NSVT.^8^ It has been reported that the prevalence of NSVT in HCM patients increases with age^8^, ^10^ and correlates with left atrial size and left ventricular (LV) wall thickness on transthoracic echocardiography (TTE),^8^, ^10^ global longitudinal peak strain, LV twist deformation and mechanical dispersion on speckle tracking echocardiography,^11^, ^12^ both the presence and extent of late gadolinium enhancement (LGE) on cardiac magnetic resonance (CMR),^13^, ^14^ as well as high heterogeneity of myocardial blood flow detected by positron emission tomography.^15^ However, there is a paucity of data on clinically accessible biomarkers and their potential prognostic values for NSVT in the setting of HOCM.

LVOT obstruction is a hallmark of more severe symptoms and worse prognosis in patients with HCM.^16^, ^17^ Previous studies indicated that circulating biomarkers representing myocardial injury (cardiac troponin I, cTnI), cardiac function (N‐terminal pro‐brain natriuretic peptide, NT‐pro BNP), and inflammation (high sensitivity C‐reactive protein, hs‐CRP) played important roles in predicting adverse outcomes in HCM patients.^18^, ^19^, ^20^ Whether these cardiac biomarkers are relevant to ventricular arrhythmias (particularly to the occurrence of NSVT) in patients with LVOT obstruction remains unresolved. Therefore, in this study, we attempt to explore and clarify the correlations between the presence of NSVT and circulating biomarkers of cTnI, NT‐pro BNP, and hs‐CRP in a relatively large Chinese HOCM cohort.

## 2 METHODS

### 2.1 Study population

We thoroughly reviewed the medical records of consecutive HOCM patients in our hospital from January 2014 to June 2019. Circulating levels of cTnI, NT‐pro BNP, and hs‐CRP, as well as cardiac evaluations of 12‐lead electrocardiogram, 24‐hour Holter monitoring, TTE, and CMR, were examined routinely in all individuals. Participants met the following diagnostic criteria of HOCM: (a) a maximum LV wall thickness ≥ 15 mm in adults (or 13‐14 mm with a definite family history of HCM) by TTE or CMR, in the absence of other accountable cardiac or systemic diseases; and (b) an instantaneous peak Doppler LVOT gradient (LVOTG) ≥30 mm Hg at rest or during physiological provocation such as Valsalva maneuver, standing, or exercise.^10^ Patients who had myocardial infarctions, myocarditis, congenital heart diseases, primary valvular heart diseases, pulmonary heart diseases, severe renal impairments, connective tissue diseases, infections, neoplasms, or with a medical history of coronary revascularization, percutaneous alcohol septal ablation, or surgical septal myectomy in the past were excluded from the study. Finally, a total of 755 HOCM patients were enrolled. The current study was in accordance with the Declaration of Helsinki, and was approved by the Ethics Committee of our hospital.

### 2.2 Laboratory examinations

Circulating levels of cTnI, NT‐pro BNP, and hs‐CRP were measured prior to all invasive procedures or treatment, when heart failure symptoms of patients could be controlled by regular oral medications. The time intervals between the blood tests of biomarkers and the completion of cardiac evaluations (Holter monitoring, TTE, and CMR) were usually within 7 days. Serum cTnI was determined using the immunochemiluminometric assay (Access AccuTnI, Beckman Coulter, California) on a Beckman Coulter Access 2 analyzer. The upper limit of its normal range (the 99th percentile of normal population) was 0.04 ng/mL, and the lower limit of detection was 0.01 ng/mL. Plasma NT‐pro BNP was examined by the electrochemiluminescent immunoassay (Elecsys pro‐BNP II assay, Roche Diagnostics, Mannheim, Germany) on a Cobas 6000 analyzer (Roche Diagnostics), with the lower detection limit of 0.6 PMol/L. A particle‐enhanced immunoturbidimetric assay (Ultrasensitive CRP kit, Orion Diagnostica, Espoo, Finland) was applied for the measurement of serum hs‐CRP on an Olympus AU‐5400 analyzer (Olympus Diagnostics). The lower detection limit of hs‐CRP was 0.25 mg/L.

### 2.3 24‐hour Holter monitoring

Twenty four‐hour Holter monitoring was tested within 3 days after the detection of circulating biomarkers. The occurrence and hourly frequency of ventricular arrhythmic events were recorded. NSVT was defined as an episode of ≥3 consecutive ventricular beats with a rate of ≥120 beats per minute, lasting <30 seconds.^10^

### 2.4 CMR image acquisition

CMR studies were performed using a 1.5‐Tesla scanner. Cine images consisting of LV two‐chamber and four‐chamber long‐axis views, LV short‐axis views, and LVOT views were acquired through true fast imaging with a steady‐state precession sequence. The maximal LV wall thickness was traced and measured from LV short‐axis views at end‐diastole. Approximately 10 to 15 minutes after a bolus injection of 0.2 mmol/kg gadolinium‐diethylenetriamine pentaacetic acid, LGE images were obtained using a segmented phase‐sensitive inversion recovery (PSIR) turbo fast low angle shot (FLASH) sequence.

### 2.5 TTE evaluation

The parasternal acoustic window was applied to record two‐dimensional and M‐mode images of left atrial diameter, LV end‐diastolic diameter and thickness of the interventricular septum. The maximum LV wall thickness was defined as the greatest thickness in any single segment of the ventricle. LV ejection fractions were calculated using the modified Simpson's rule method. Severity degrees of mitral regurgitation were assessed semiquantitatively by the color Doppler flow imaging. The LVOTGs at rest were measured using pulsed and continuous‐wave Doppler from the apical three and five chamber views. The provoked LVOTGs were detected by the Valsalva maneuver, standing, or exercise.

### 2.6 SCD risk estimation

The risk of SCD in HOCM patients was predicted using the ESC online HCM risk‐SCD calculator. According to this risk model, patients with an HCM Risk‐SCD higher than 6% were considered as high risk.^7^, ^10^

### 2.7 Statistical analysis

Categorical variables were compared using chi‐square tests or Fisher's exact tests. Continuous variables were compared using unpaired Student's *t* tests or nonparametric tests. Kruskal‐Wallis H tests were performed to compare the differences among multiple independent samples in nonparametric tests. The correlations between two continuous variables were determined using Pearson's correlation tests. Univariable and multivariable logistic regression analyses were applied to identify independent indexes associated with NSVT in the HOCM cohort. Due to the skewed distribution of cTnI, NT‐pro BNP, and hs‐CRP, they were converted into natural logarithmic transformations for *t* tests, correlation tests, and regression analyses. The area under the curve (AUC), optimal cutoff values, sensitivity and specificity of cTnI in predicting NSVT were determined using the receiver‐operating characteristic (ROC) curve analysis. The statistical package SPSS 22.0 (SPSS Inc., Chicago, Illinois) was applied for all statistical analyses. A two‐tailed *P*‐value of <.05 was considered as statistically significant.

## 3 RESULTS

### 3.1 Demographics, clinical features, and medications of the HOCM cohort

Our study cohort consisted of 755 consecutive HOCM patients, with 57.4% males and a mean age of 51.0 ± 12.9 years. A total of 138 patients (18.3%) with positive NSVT episodes on Holter monitoring were classified into the NSVT group, and the rest 617 patients with negative VT detection were categorized as the nonventricular tachycardia (non‐VT) group. Although patients diagnosed with NSVT seemed to have comparatively more complaints of chest pain, dyspnea, palpitation, and unexplained syncope, the statistical differences between the two groups were unremarkable. Hypertension accounted for a greater proportion in individuals of the non‐VT group (*P* = .029). Beta‐blockers were prescribed more frequently in HOCM patients with NSVT (*P* = .040) (Table 1).

**TABLE 1:** Demographics, clinical features, and medical treatments of the HOCM cohort

	Total population (n = 755)	NSVT group (n = 138)	Non‐VT group (n = 617)	*P*‐value
Age (years)^a^	51.0 ± 12.9	50.9 ± 13.1	51.0 ± 12.9	.914
Male, n (%)	433 (57.4%)	79 (57.2%)	354 (57.4%)	.978
Chest pain, n (%)	482 (63.8%)	92 (66.7%)	390 (63.2%)	.445
Dyspnea, n (%)	606 (80.3%)	117 (84.8%)	489 (79.3%)	.140
Palpitation, n (%)	269 (35.6%)	50 (36.2%)	219 (35.5%)	.870
Syncope, n (%)^a^	179 (23.7%)	39 (28.3%)	140 (22.7%)	.164
Hypertension, n (%)	280 (37.1%)	40 (29.0%)	240 (38.9%)	.029
Diabetes mellitus, n (%)	56 (7.4%)	7 (5.1%)	49 (7.9%)	.245
Hyperlipidemia, n (%)	260 (34.4%)	40 (29.0%)	220 (35.7%)	.136
Current smokers, n (%)	286 (37.9%)	51 (37.0%)	235 (38.1%)	.804
Alcohol drinking, n (%)	140 (18.5%)	22 (15.9%)	118 (19.1%)	.384
FH of HCM, n (%)^a^	78 (10.3%)	17 (12.3%)	61 (9.9%)	.396
FH of SCD, n (%)	40 (5.3%)	6 (4.3%)	34 (5.5%)	.581
SBP (mm Hg)	123.3 ± 16.7	121.9 ± 16.1	123.6 ± 16.8	.298
DBP (mm Hg)	74.0 ± 10.2	73.4 ± 10.7	74.2 ± 10.1	.432
HR (beats/min)	68.0 ± 10.1	68.6 ± 10.4	67.8 ± 10.0	.411
BMI (kg/m^2^)	25.7 ± 3.4	25.2 ± 4.0	25.9 ± 3.3	.098
NYHA heart function class				
Class I, n (%)	115 (15.2%)	19 (13.8%)	96 (15.6%)	.597
Class II, n (%)	397 (52.6%)	77 (55.8%)	320 (51.9%)	.403
Class III, n (%)	232 (30.7%)	41 (29.7%)	191 (31.0%)	.774
Class IV, n (%)	11 (1.5%)	1 (0.7%)	10 (1.6%)	.699
Medications				
Beta‐blockers, n (%)	496 (65.7%)	101 (73.2%)	395 (64.0%)	.040
Calcium antagonists, n (%)	182 (24.1%)	36 (26.1%)	146 (23.7%)	.547
ACEI/ARB, n (%)	103 (13.6%)	18 (13.0%)	85 (13.8%)	.821
Statins, n (%)	128 (17.0%)	24 (17.4%)	104 (16.9%)	.880
Diuretics, n (%)	51 (6.8%)	12 (8.7%)	39 (6.3%)	.315
Aspirin, n (%)	157 (20.8%)	31 (22.5%)	126 (20.4%)	.593
Anticoagulants, n (%)	23 (3.0%)	5 (3.6%)	18 (2.9%)	.591

Abbreviations: ACEI, angiotensin‐converting enzyme inhibitor; ARB, angiotensin receptor blocker; BMI, body mass index; DBP, diastolic blood pressure; FH, family history; HCM, hypertrophic cardiomyopathy; HOCM, hypertrophic obstructive cardiomyopathy; HR, heart rate; NSVT, nonsustained ventricular tachycardia; NYHA, New York Heart Association; SBP, systolic blood pressure; SCD, sudden cardiac death; VT, ventricular tachycardia.

^a^ Items included in the HCM risk‐SCD calculator.

### 3.2 Comparisons of circulating biomarkers and cardiac evaluations between the NSVT and the non‐VT group

**FIGURE 1:**
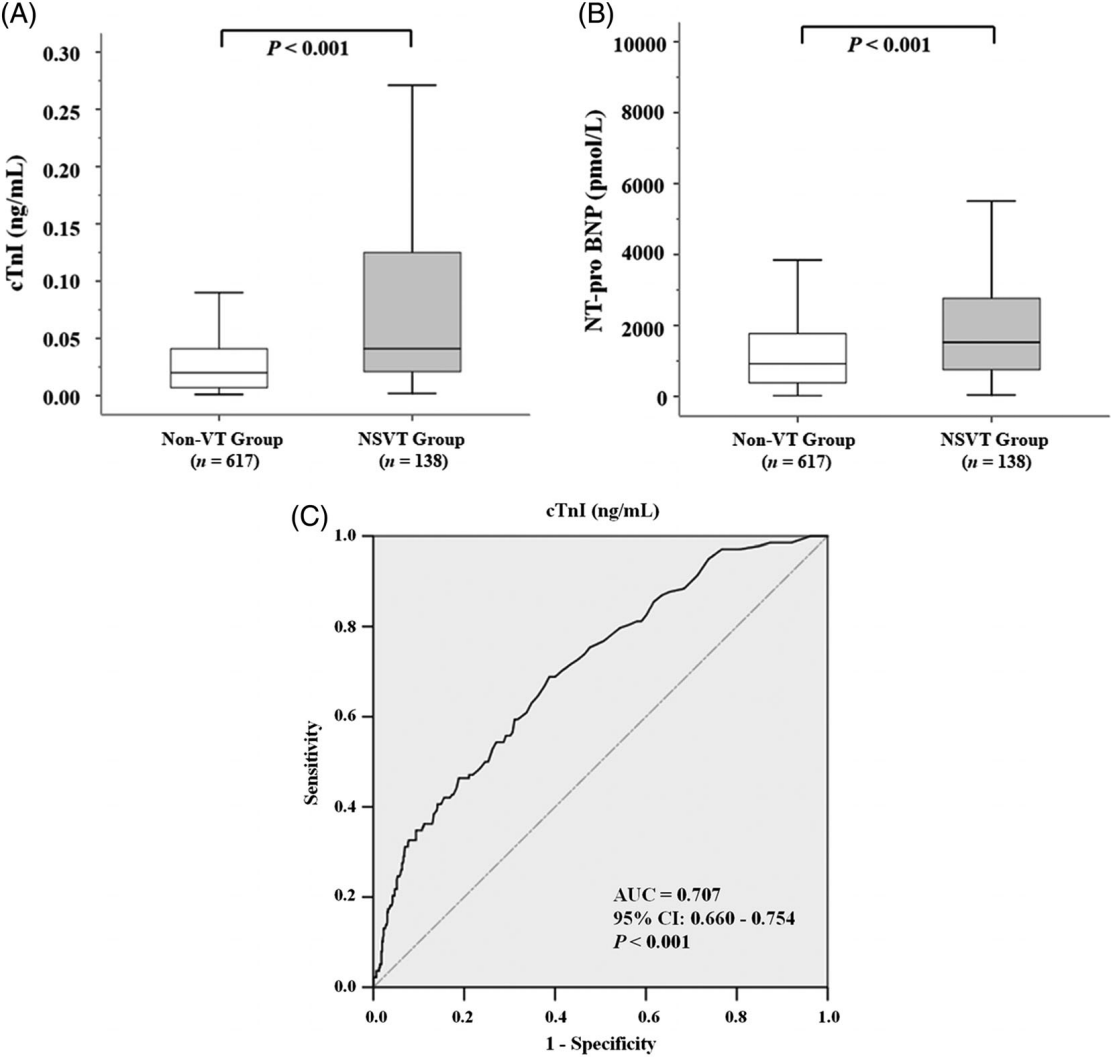
Circulating levels of A, cTnI and B, NT‐pro BNP in HOCM patients with or without NSVT. C, The ROC curve of cTnI to predict the presence of NSVT in the HOCM cohort. AUC, area under the curve; CI, confidence interval; cTnI, cardiac troponin I; HOCM, hypertrophic obstructive cardiomyopathy; NSVT, nonsustained ventricular tachycardia; NT‐pro BNP, N‐terminal pro‐brain natriuretic peptide; ROC, receiver‐operating characteristic

Patients in the NSVT group had significantly elevated levels of cTnI (*P* < .001) and NT‐pro BNP (*P* < .001), whereas the concentrations of serum hs‐CRP were similar between the two groups (Table 2, Figure 1A,B). Holter monitoring revealed that patients with NSVT suffered from increased burden of ventricular arrhythmic events, with greater numbers of premature ventricular contractions (PVCs) (*P* = .001), more various PVC morphologies (*P* < .001), as well as higher prevalence of ventricular bigeminy (*P* < .001), and paired ventricular beats (*P* < .001). CMR indicated that NSVT patients possessed much thicker ventricular walls (*P* < .001), slightly decreased LV ejection fractions (*P* = .018), and enhanced positive rate of LGE (*P* = .003). TTE showed not significant differences either in peak LVOTGs or in severity degrees of mitral regurgitation between the two groups (Table 2). In our study, 86.2% of the entire population presented with positive LGE on CMR, and we further analyzed the involved locations of LGE in our HOCM cohort (Table S1). Compared to patients with LGE located only in the interventricular septal area, the proportion of NSVT in patients with LGE in both septum and other ventricular walls was significantly higher (22.8% vs 13.7%, *P* = .007).

**TABLE 2:** Comparisons of circulating biomarkers and cardiac evaluations between the NSVT and the non‐VT group

	Total population (n = 755)	NSVT group (n = 138)	Non‐VT group (n = 617)	*P‐*value
Blood tests				
cTnI (ng/mL)	0.022 (0.008‐0.047)	0.041 (0.021‐0.129)	0.020 (0.007‐0.041)	<.001
NT‐pro BNP (pmol/L)	1022.0 (447.7‐2014.0)	1525.0 (738.1‐2772.8)	923.0 (383.5‐1776.5)	<.001
hs‐CRP (mg/L)	1.130 (0.550‐2.310)	1.165 (0.598‐2.910)	1.120 (0.530‐2.260)	.553
ALT (iu/l)	21.0 (15.0‐30.0)	19.0 (15.0‐31.0)	21.0 (15.0‐30.0)	.412
Cr (μmol/L)	77.0 (67.7‐86.7)	78.3 (68.4‐87.6)	77.0 (67.5‐86.6)	.578
Glu (mmol/L)	4.88 (4.42‐5.34)	4.84 (4.39‐5.27)	4.89 (4.43‐5.35)	.384
24‐hour Holter monitoring				
Total PVCs (beats)	358.2 ± 1975.0	1370.7 ± 4257.6	131.8 ± 679.7	.001
Maximum PVCs/h (beats)	46.3 ± 196.4	170.9 ± 401.5	18.4 ± 84.4	<.001
Polymorphic PVC, n (%)	431 (57.1%)	114 (82.6%)	317 (51.4%)	<.001
PVC morphology (types)	1.8 ± 1.3	2.8 ± 1.3	1.6 ± 1.2	<.001
Ventricular bigeminy, n (%)	111 (14.7%)	49 (35.5%)	62 (10.0%)	<.001
Paired PVC, n (%)	192 (25.4%)	85 (61.6%)	107 (17.3%)	<.001
NSVT, n (%)^a^	138 (18.3%)	138 (100%)	0	—
Atrial fibrillation, n (%)	146 (19.3%)	31 (22.5%)	115 (18.6%)	.304
CMR				
LAD (mm)	42.1 ± 8.4	43.0 ± 9.0	41.9 ± 8.3	.176
LVEDD (mm)	45.6 ± 4.7	45.8 ± 5.3	45.6 ± 4.5	.620
MWT (mm)	24.0 ± 5.2	25.8 ± 5.2	23.6 ± 5.1	<.001
IVS≥30 mm, n (%)	118 (15.6%)	37 (26.8%)	81 (13.1%)	.001
LVEF (%)	65.5 ± 7.5	64.0 ± 8.6	65.9 ± 7.1	.018
CO (L/min)	6.3 ± 3.3	6.7 ± 5.5	6.2 ± 2.6	.263
LVEDV (mL)	139.8 ± 38.4	146.0 ± 43.3	138.4 ± 37.1	.060
LGE(+), n (%)	651 (86.2%)	130 (94.2%)	521 (84.4%)	.003
TTE				
LAD on TTE (mm)^a^	43.4 ± 7.0	44.8 ± 7.3	43.1 ± 7.0	.011
MWT on TTE (mm)^a^	22.1 ± 5.0	23.9 ± 5.3	21.7 ± 4.8	<.001
Peak LVOT flow velocity (m/s)	4.43 ± 0.83	4.43 ± 0.70	4.43 ± 0.86	.994
Peak LVOTG (mm Hg)^a^	81.3 ± 29.5	80.4 ± 25.5	81.5 ± 30.3	.672
Mitral regurgitation, n (%)				
Absent, n (%)	23 (3.0%)	5 (3.6%)	18 (2.9%)	.591
Mild, n (%)	236 (31.3%)	34 (24.6%)	202 (32.7%)	.063
Moderate, n (%)	374 (49.5%)	75 (54.3%)	299 (48.5%)	.211
Severe, n (%)	122 (16.2%)	24 (17.4%)	98 (15.9%)	.664
Risk of SCD at 5 y (%)	4.4 ± 3.1	8.6 ± 4.2	3.5 ± 1.8	<.001

Abbreviations: ALT, alanine aminotransferase; CMR, cardiac magnetic resonance; CO, cardiac output; Cr, serum creatinine; cTnI, cardiac troponin I; Glu, glucose; HOCM, hypertrophic obstructive cardiomyopathy; hs‐CRP, high‐sensitivity C‐reactive protein; IVS, interventricular septum; LAD, left atrial diameter; LGE(+), positive late gadolinium enhancement; LVEDD, left ventricular end‐diastolic diameter; LVEDV, left ventricular end‐diastolic volume; LVEF, left ventricular ejection fraction; LVOT, left ventricular outflow tract; LVOTG, left ventricular outflow tract gradient; MWT, maximum wall thickness; NSVT, nonsustained ventricular tachycardia; NT‐pro BNP, N‐terminal pro‐brain natriuretic peptide; PVC, premature ventricular contraction; SCD, sudden cardiac death; TTE, transthoracic echocardiography.

^a^ Items included in the HCM risk‐SCD calculator.

### 3.3 Logistic regression analyses to identify independent determinants of NSVT

Univariable logistic regression analyses suggested that Ln cTnI, Ln NT‐pro BNP, maximal LV wall thickness, LV ejection fraction, LV end‐diastolic volume, and LGE(+) were significantly associated with the occurrence of NSVT in HOCM patients (Table 3). All the covariates with *P*‐value < .05 in univariable analyses were further included in the multivariable model. After adjusting for all relevant variables, a strong independent correlation was demonstrated between Ln cTnI and the presence of NSVT in the HOCM cohort (OR = 1.675, 95%CI: 1.406‐1.994, *P* < .001) (Table 3).

**TABLE 3:** Logistic regression analyses to identify independent determinants of NSVT in HOCM

Total HOCM population (n = 755)			
Univariable logistic regression analysis	OR	95% CI	*P*‐value
Age (years)	0.999	0.985‐1.014	.914
Male, n (%)	0.995	0.685‐1.445	.978
BMI (kg/m^2^)	0.949	0.899‐1.002	.058
NYHA III or IV, n (%)	0.905	0.607‐1.351	.626
Ln cTnI (ng/mL)	1.828	1.565‐2.136	<.001
Ln NT‐pro BNP (pmol/L)	1.557	1.299‐1.866	<.001
Ln hs‐CRP (mg/L)	1.030	0.911‐1.166	.636
LAD (mm)	1.015	0.993‐1.038	.176
LVEDD (mm)	1.011	0.972‐1.052	.583
MWT (mm)	1.080	1.044‐1.117	<.001
LVEF (%)	0.968	0.944‐0.991	.008
CO (L/min)	1.037	0.989‐1.086	.130
LVEDV (mL)	1.005	1.000‐1.009	.038
Peak LVOT flow velocity (m/s)	0.999	0.800‐1.247	.994
Peak LVOTG (mm Hg)	0.999	0.992‐1.005	.703
Moderate to severe MR, n (%)	1.407	0.938‐2.110	.099
LGE (+), n (%)	2.994	1.419‐6.316	.004
Multivariable logistic regression analysis	OR	95% CI	*P*‐value
Ln cTnI (ng/mL)	1.675	1.406‐1.994	<.001
Ln NT‐pro BNP (pmol/L)	1.206	0.981‐1.482	.076
MWT (mm)	1.013	0.971‐1.056	.548
LVEF (%)	0.998	0.972‐1.024	.881
LVEDV (mL)	1.000	0.995‐1.006	.878
LGE(+), n (%)	1.429	0.645‐3.168	.379

Abbreviations: BMI, body mass index; CI, confidence interval; CO, cardiac output; cTnI, cardiac troponin I; HOCM, hypertrophic obstructive cardiomyopathy; hs‐CRP, high‐sensitivity C‐reactive protein; LAD, left atrial diameter; LGE(+), positive late gadolinium enhancement; LVEDD, left ventricular end‐diastolic diameter; LVEDV, left ventricular end‐diastolic volume; LVEF, left ventricular ejection fraction; LVOT, left ventricular outflow tract; LVOTG, left ventricular outflow tract gradient; MR, mitral regurgitation; MWT, maximum wall thickness; NSVT, nonsustained ventricular tachycardia; NT‐pro BNP, N‐terminal pro‐brain natriuretic peptide; NYHA, New York Heart Association; OR, odds ratio.

### 3.4 ROC curve analysis of cTnI to predict NSVT in HOCM

The efficiency of cTnI in predicting the occurrence of NSVT was evaluated by ROC curve analysis (Figure 1C). The AUC of cTnI was 0.707 (95% CI: 0.660‐0.754, *P* < .001). The optimal cutoff value of cTnI was 0.0265 ng/mL, with the sensitivity of 68.8% and the specificity of 61.3%.

### 3.5 Levels of cTnI according to the classification of ventricular arrhythmias

**FIGURE 2:**
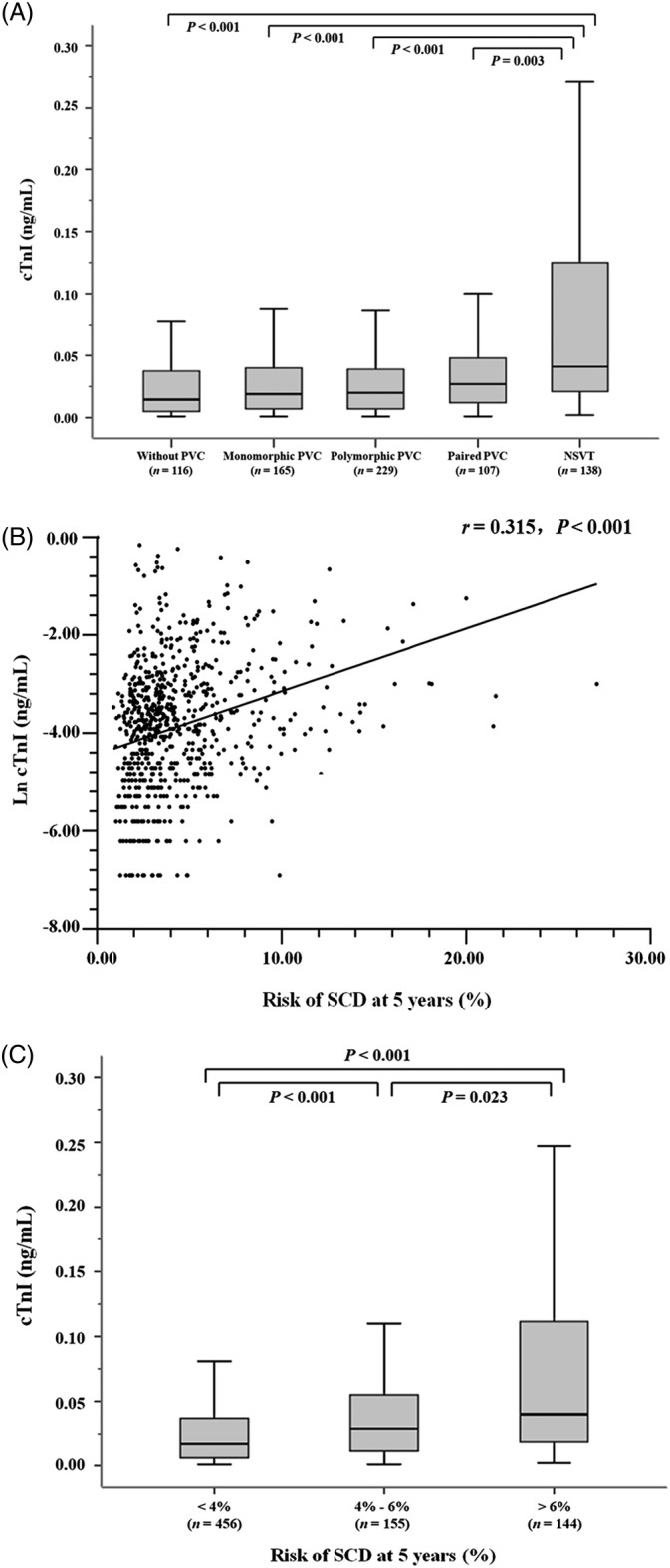
A, Levels of serum cTnI according to classifications of ventricular arrhythmic events in patients with HOCM. B,C, Correlations between cTnI and the risk of SCD at 5 years in patients with HOCM. cTnI, cardiac troponin I; HOCM, hypertrophic obstructive cardiomyopathy; NSVT, nonsustained ventricular tachycardia; PVC, premature ventricular contraction; SCD, sudden cardiac death

HOCM patients were divided into five groups according to the severity and complexity of ventricular arrhythmias on 24‐hour Holter monitoring (Figure 2A). Levels of cTnI displayed a general uptrend with the aggravation of ventricular arrhythmic events in HOCM (*P* < .001). Concentrations of cTnI in the NSVT group were significantly higher than their counterparts in the non‐PVC group (*P* < .001), the monomorphic PVC group (*P* < .001), the polymorphic PVC group (*P* < .001), and the paired PVC group (*P* = .003), respectively.

### 3.6 Correlations between cTnI and the risk of SCD in the HOCM cohort

Correlation analyses indicated that Ln cTnI was positively related to the 5‐year risk of SCD in the HOCM cohort (*r* = 0.315, *P* < .001) (Figure 2B). Moreover, compared with individuals in the low (5‐year risk <4%) or intermediate (5‐year risk within 4%‐6%) risk groups, concentrations of cTnI elevated significantly in patients in the high‐risk group of SCD (5‐year risk >6%) (Figure 2C).

## 4 DISCUSSION

Cardiac troponins, natriuretic peptides and C‐reactive proteins, as specific and sensitive biomarkers of myocardial damage, cardiac failure, and inflammation, are well‐established tools for the diagnosis and prognosis in various cardiovascular conditions. Accumulating evidence has shown that elevated baseline levels of cTnI, NT‐pro BNP, and hs‐CRP have played important roles in predicting mortality and combined adverse outcomes in HCM.^18^, ^19^, ^20^ However, only sparse data are available concerning the correlations between these cardiac biomarkers and the presence of ventricular arrhythmias in patients with HOCM. The major results of our study suggested that circulating levels of both cTnI and NT‐pro BNP increased significantly in patients with positive NSVT episodes, but only serum cTnI was independently associated with the occurrence of NSVT and the complexity of ventricular arrhythmic events in our large HOCM cohort.

Cardiac troponins are demonstrated to be elevated in patients with HCM. It has been reported that higher concentrations of troponins correlate with age, male gender, left atrial diameter, maximal LV wall thickness, LVOTG, myocardial fibrosis, NYHA functional class, and prevalence of atrial fibrillation in affected patients.^18^, ^21^, ^22^, ^23^, ^24^ Serum cTnI is closely related to increased occurrence of ventricular arrhythmias and can identify a subgroup of patients with VT in the setting of chronic heart failure.^25^ In a previous study exploring the condition of myocardial ischemia diagnosed by the release of high sensitivity‐TnI (hs‐TnI) in HCM, NSVT episode was detected in 75% of hs‐TnI positive patients, and elevated hs‐TnI levels were associated with higher frequency of NSVT.^26^ As for natriuretic peptides, numerous cross‐sectional studies have shown that plasma BNP concentrations are increased in patients with HCM and related to symptoms of heart failure, exercise capacity, severity of hypertrophy, LVOT obstruction, LV systolic and diastolic dysfunctions, left atrial diameter, LV mass index, and LGE(+) detected by CMR.^19^, ^27^, ^28^, ^29^, ^30^, ^31^, ^32^, ^33^, ^34^ A relatively small‐scale clinical investigation on HCM demonstrated a remarkable dispersion of NT‐pro BNP levels between patients with and without NSVT.^35^ In accordance with previous studies, we identified significantly elevated levels of both cTnI and NT‐pro BNP in HOCM patients with NSVT. Moreover, multivariable analyses revealed that cTnI was the only independent determinant of NSVT in the setting of HOCM. Furthermore, serum cTnI ≥ 0.0265 ng/mL indicated predictive value for the presence of NSVT in our HOCM cohort.

Different mechanisms may account for the association between elevated cTnI and ventricular arrhythmias in patients with HOCM. This cardiac disorder is characterized pathologically by cardiomyocyte hypertrophy, myofibrillar disarray, and interstitial fibrosis, resulting in the disorganized LV myocardial architecture in affected patients.^2^ Hypertrophied myocardium usually calls for increased demand of oxygen and blood. The imbalance between inappropriate hypertrophy of the myocardium and insufficient coronary arterial supply, particularly under the condition of LVOT obstruction, will give rise to a complex interplay of elevated wall stress, prolonged ventricular relaxation, enhanced LV filling pressure and reduced myocardial perfusion reserve, lead to microvascular dysfunction, myocardial ischemia and myocyte necrosis, and finally result in the release of cardiac troponins.^36^, ^37^, ^38^ In addition, myocardial disarray and fibrosis, as well as myocardial replacement scarring in HOCM can serve as potentially arrhythmogenic substrates of life‐threatening electrical instability.^2^ Areas of myocardial fibrosis interspersed with normal cardiomyocytes will form regions of conduction block that may promote the dispersion of electrical depolarization and repolarization, and induce re‐entry circuits leading to increased susceptibility to ventricular arrhythmias.^39^ Furthermore, the frequent contractions in highly abnormal patterns of ventricular arrhythmias, in turn, can cause mechanical stress upon the myocardium and further deteriorate myocardial ischemia, which provides another important explanation for the biochemically detected myocardial injury in this setting.^40^, ^41^ As shown in our study, patients of the NSVT group presented with more severe ventricular hypertrophy, lower LV ejection fraction, and increased myocardial fibrosis assessed by LGE in CMR. These manifestations in morphology and hemodynamics were quite consistent with the abovementioned mechanisms between elevated cTnI and ventricular arrhythmias in HOCM patients. Interestingly, besides the strong association with NSVT, we also found that the concentration of cTnI significantly correlated with increased risks for both frequent PVCs and complex ventricular arrhythmias such as ventricular bigeminy, polymorphic PVCs, and paired PVCs in HOCM patients. The level of cTnI elevated progressively with the growing severity and complexity of ventricular arrhythmic events in our cohort, suggesting its potential value in assessing the detailed clinical status of ventricular arrhythmias in patients with HOCM. Furthermore, analysis by CMR also roughly indicated that the more locations of LGE in the ventricular walls, the higher proportion of NSVT might be in patients with HOCM. However, more standard and accurate quantification of LGE is required in future studies.

SCD is perhaps the most unpredictable and devastating consequence of HCM. Although the current HCM risk‐SCD calculator is a practical scoring model developed to identify high‐risk patients and has been validated in adults, the prediction of SCD in HCM still remains challenging due to the heterogeneity of clinical expressions and the relatively low event rate observed in this disease. Apart from traditional risk factors, some novel imaging parameters on CMR have been recently studied for prognostic values as risk stratifiers of HCM.^42^, ^43^ However, cardiac evaluations by CMR are relatively high in cost, and are not so widely available in common medical institutions. In this context, exploring more easily applicable predictive factors for SCD is of great clinical significance. In our study, we found a positive correlation between Ln cTnI and the 5‐year risk of SCD in HOCM. Individuals with a HCM risk‐SCD over 6% had significantly elevated serum cTnI levels compared with their counterparts in the low or intermediate risk groups. Our results indicated, to some extent, that higher concentrations of cTnI might be helpful in the pre‐evaluation for the risk of SCD in HOCM. Since the measurement of cTnI is simple and widely accessible, it should be used as part of a routine assessment to provide more screening and prognostic information for the clinical status of ventricular arrhythmias and the risk of SCD in patients with HOCM. However, to further demonstrate whether cTnI is additive to the risk prediction of HOCM patients, more long‐term prospective follow‐up studies are needed in the future.

### 4.1 Study limitations

First, this was a single‐center, cross‐sectional retrospective study. Although we suggested an independent association between elevated levels of cTnI and the presence of NSVT in HOCM patients, the retrospective nature of the current study limited our ability to determine a causal relationship. Second, the positive detection rate of NSVT episodes was based on the results of 24‐hour Holter monitoring. The relatively short duration of the examination might have somehow underestimated the incidence of NSVT in the HOCM cohort. Finally, the risk of SCD in the study population was estimated by the HCM risk‐SCD score due to the absence of long‐term clinical follow‐up data. There was no doubt that prospective studies were desirable; however, since a considerable number of subjects underwent alcohol septal ablation or septal myectomy in their later course of the disease, those invasive procedures could inevitably affect the natural prevalence of NSVT and the outcomes of HOCM patients. Therefore, we chose to utilize the scoring model to estimate the risk of SCD in our HOCM cohort.

## 5 CONCLUSIONS

To the best of our knowledge, this was the first study that systematically investigated correlations of different circulating biomarkers (cTnI, NT‐pro BNP, and hs‐CRP) and the presence of NSVT in a large Chinese HOCM cohort. Elevated serum cTnI was an independent determinant of NSVT episodes, and it seemed to be a valuable index to assess the clinical status of ventricular arrhythmias and the risk of SCD in patients with HOCM.

## CONFLICT OF INTEREST

The authors declare no potential conflict of interests.

## Supporting information


**Table S1** Locations of LGE in HOCM patients with or without NSVT.
