# Supplementary material for: Correlations between cardiac troponin I and nonsustained ventricular tachycardia in hypertrophic obstructive cardiomyopathy
Source: Clin Cardiol. 2020 Aug 18;43(10):1150–9. doi: 10.1002/clc.23425 (PMC7534013; doi:10.1002/clc.23425)
Supplement: Supplementary file 1 — Table S1 Locations of LGE in HOCM patients with or without NSVT. [file CLC-43-1150-s001.docx]

**Supplementary Table 1 Locations of LGE in HOCM patients with or without NSVT**

| Locations of LGE | Total Population  (*n* = 651) | NSVT Group  (*n* = 130) | Non-VT Group  (*n* = 521) | *P*-value |
| --- | --- | --- | --- | --- |
| interventricular septum, *n* (%) | 613 (94.2%) | 122(93.8%) | 491(94.2%) | 0.863 |
| Anterior wall of lv, *n* (%) | 299(45.9%) | 73(56.2%) | 226(43.4%) | 0.009 |
| Inferior wall of LV, *n* (%) | 301(46.2%) | 73(56.2%) | 228(43.8%) | 0.011 |
| Lateral wall of lv, *n* (%) | 62(9.5%) | 15(11.5%) | 47(9.0%) | 0.382 |
| Posterior wall of LV, *n* (%) | 35(5.4%) | 10(7.7%) | 25(4.8%) | 0.191 |
| apex of LV, *n* (%) | 58(8.9%) | 19(14.6%) | 39(7.5%) | 0.011 |
| Right ventricle, *n* (%) | 15(2.3%) | 5(3.8%) | 10(1.9%) | 0.190 |
| Papillary muscle, *n* (%) | 71(10.9%) | 10(7.7%) | 61(11.7%) | 0.189 |

HOCM indicates hypertrophic obstructive cardiomyopathy; NSVT, nonsustained ventricular tachycardia; LGE, late gadolinium enhancement; and LV, left ventricle.
